# A Systematic Study of the Stability, Safety, and Efficacy of the *de novo* Designed Antimicrobial Peptide PepD2 and Its Modified Derivatives Against *Acinetobacter baumannii*

**DOI:** 10.3389/fmicb.2021.678330

**Published:** 2021-06-18

**Authors:** Sung-Pang Chen, Eric H-L Chen, Sheng-Yung Yang, Pin-Shin Kuo, Hau-Ming Jan, Tsai-Chen Yang, Ming-Yen Hsieh, Kung-Ta Lee, Chun-Hung Lin, Rita P-Y Chen

**Affiliations:** ^1^Institute of Biological Chemistry, Academia Sinica, Taipei, Taiwan; ^2^Institute of Biochemical Sciences, National Taiwan University, Taipei, Taiwan; ^3^Department of Biochemical Science and Technology, National Taiwan University, Taipei, Taiwan

**Keywords:** antimicrobial peptide, antibiotic-resistant, *Acinetobacter baumannii*, lipid, membrane, drug-resistant

## Abstract

Searching for new antimicrobials is a pressing issue to conquer the emergence of multidrug-resistant (MDR) bacteria and fungi. Antimicrobial peptides (AMPs) usually have antimicrobial mechanisms different from those of traditional antibiotics and bring new hope in the discovery of new antimicrobials. In addition to antimicrobial activity, stability and target selectivity are important concerns to decide whether an antimicrobial peptide can be applied *in vivo*. Here, we used a simple *de novo* designed peptide, pepD2, which contains only three kinds of amino acid residues (W, K, L), as an example to evaluate how the residues and modifications affect the antimicrobial activity against *Acinetobacter baumannii*, stability in plasma, and toxicity to human HEK293 cells. We found that pepI2 with a Leu→Ile substitution can decrease the minimum bactericidal concentrations (MBC) against *A. baumannii* by one half (4 μg/mL). A D-form peptide, pepdD2, in which the D-enantiomers replaced the L-enantiomers of the Lys(K) and Leu(L) residues, extended the peptide half-life in plasma by more than 12-fold. PepD3 is 3-residue shorter than pepD2. Decreasing peptide length did not affect antimicrobial activity but increased the IC_50_ to HEK293 cells, thus increased the selectivity index (SI) between *A. baumannii* and HEK293 cells from 4.7 to 8.5. The chain length increase of the N-terminal acyl group and the Lys→Arg substitution greatly enhanced the hemolytic activity, hence those modifications are not good for clinical application. Unlike colistin, the action mechanism of our peptides relies on negatively charged lipids rather than lipopolysaccharides. Therefore, not only gram-negative bacteria but also gram-positive bacteria can be killed by our peptides.

## Introduction

Over the last three decades, many microbial isolates resistant to penicillins, cephalosporins, carbapenems, fluoroquinolones, and aminoglycosides have been reported. The emergence of multidrug-resistant (MDR) bacteria and fungi is a severe threat to global human health. In 2017, the WHO published a warning list of twelve antibiotic-resistant “priority bacteria families” that have an urgent need for new antibiotics. During the recent COVID-19 pandemic, the widespread use of broad-spectrum antimicrobials and corticosteroids to treat patients, the use of mechanical ventilation and prolonged hospitalization might increase the risk for MDR pathogen infection. Antimicrobial peptides (AMPs) have antimicrobial mechanisms different from pre-existing small molecule antibiotics; hence, they can effectively eliminate MDR pathogens and become potential alternatives to combat superbugs (Hancock and Sahl, 2006; Deslouches et al., 2013; Feng et al., 2013; Mahlapuu et al., 2016; Lei et al., 2019; Mohan et al., 2019; Raheem and Straus, 2019; Magana et al., 2020; Neshani et al., 2020; Upert et al., 2020). Colistin, a peptide antibiotic against Gram-negative bacteria, was discovered in the 1940s. Due to the emergence of MDR bacteria, polymyxins (colistin and its derivatives) are now clinically used as the last-line therapy for drug-resistant bacterial infection despite the possibility of colistin-induced acute kidney injury (Evans et al., 1999; Falagas et al., 2005; Falagas and Kasiakou, 2006; Zavascki et al., 2007; Lim et al., 2010; Gai et al., 2019; Yousfi et al., 2019). As a polycationic peptide, colistin targets lipopolysaccharide (LPS) in the outer membrane of Gram-negative bacteria, displaces divalent cations (Ca^2+^ and Mg^2+^), changes membrane permeability, and disrupts the bacterial membrane. However, colistin resistance rapidly emerged by a change in the lipid composition in the bacterial membrane to reduce its binding affinity with colistin (Bialvaei and Samadi Kafil, 2015).

Natural AMPs are a self-defense mechanism of organisms. In addition to antimicrobial activity, other functions, such as antibiofilm, immunomodulation, anticancer, and anti-inflammatory activities, have been reported for AMPs. They can be linear or cyclized and in an α-helix, β-sheet, α/β mixed form, or random coil structure (Bahar and Ren, 2013; Wang, 2015; Mahlapuu et al., 2016; Kumar et al., 2018). The net charge of AMPs could range from −12 to +30, and the hydrophobic content has a broad range as well (from 0 to 93%). Many AMPs were designed *in cerebro* or *in silico* based on natural AMPs (Blondelle and Houghten, 1991; Fjell et al., 2011; Luong et al., 2020). Among them, a class of cationic, amphiphilic, and α-helical peptides have received much attention. Many AMPs in this class were designed and showed excellent antimicrobial efficacy against Gram-positive and Gram-negative bacteria (Blondelle and Houghten, 1992; Papo et al., 2002; Deslouches et al., 2005, 2013). Moreover, because of the rapid direct membrane-disruption mechanism of AMPs, the development of resistance to AMPs is less likely (Mohan et al., 2019).

*Acinetobacter baumannii*, a rod-shaped Gram-negative bacterium, has been ranked in the first tier in the WHO warning list. As an opportunistic pathogen, it is one of the leading causes of nosocomial infections worldwide (Almasaudi, 2018). Recently, several designed peptides have shown promising efficiency in eliminating *A. baumannii* in mice (Neshani et al., 2020). An *in silico* designed 20-mer peptide, named Ω76, showed high efficacy against carbapenem- and tigecycline-resistant *A. baumannii* in a mouse intraperitoneal infection model (Nagarajan et al., 2019). A cyclic peptide, ZY4 (17-mer), redesigned and based on cathelicidin-BF, had low toxicity and can suppress the dissemination of *A. baumannii* to target organs (Mwangi et al., 2019). LysAB2 P3 (33-mer) was modified from the C-terminal segment of the *A. baumannii* phage endolysin LysAB2. It showed no toxicity against normal eukaryotic cells, and its minimum bactericidal concentrations (MBC) against standard *A. baumannii* ATCC 17978, 19606 and clinically isolated colistin-resistant MDR *A. baumannii* were 8 μM (approximately 30 μg/mL). A single intraperitoneal injection of LysAB2 P3 (7.5 mg/kg) 3 h after infection can rescue 60% of the mice from *A. baumannii* infection (Peng et al., 2017).

Recently, we *de novo* designed a peptide named pepD2 with the sequence WKKLKKLLKKLKKL, which has a trigonal distribution of positive charges when a helical structure is formed (unpublished data). Since many proteases have preferred recognition residues or sequences, we simply used only three residues in this peptide. Trp was used for peptide identification and quantification. Lys was chosen as the positively charged residue due to cost concerns. Leu was used since Leu favors helix formation. The N-terminus of the peptide is acetylated, and the C-terminus is amidated to avoid digestion by exopeptidases *in vivo*. Using this peptide as a simple system and *A. baumannii* as our target pathogen, we systematically evaluated how changes in amino acid residues and end protection affected the antimicrobial effects, hemolytic activity, cytotoxicity, and stability of AMPs. The peptides used in this study are listed in Table 1.

**TABLE 1:** The antimicrobial peptides designed in this study.

Peptide	Sequence
pepD2	Ac-WKKLKKLLKKLKKL-NH_2_
pepD3	Ac-WKKLKKLLKKL-NH_2_
pepV2	Ac-WKKVKKVVKKVKKV-NH_2_
pepI2	Ac-WKKIKKIIKKIKKI-NH_2_
pepR2	Ac-WRRLRRLLRRLRRL-NH_2_
pepO2	Ac-WOOLOOLLOOLOOL-NH_2_
pepdD2	Ac-Wkklkkllkklkkl-NH_2_
pepD2M	Myr-WKKLKKLLKKLKKL-NH_2_
pepD2P	Pal-WKKLKKLLKKLKKL-NH_2_
pepD2S	Ste-WKKLKKLLKKLKKL-NH_2_
pepD3M	Myr-WKKLKKLLKKL-NH_2_
pepD3O	Oct-WKKLKKLLKKL-NH_2_
pepD3H	Hex-WKKLKKLLKKL-NH_2_
pepD3B	But-WKKLKKLLKKL-NH_2_

*O, ornithine; Ac, acetyl; Myr, myristyl; Pal, palmitoyl; Ste, stearyl; Oct, octanoyl; Hex, hexanoyl; But, butanoyl. The D-form amino acids are expressed as lowercase letters.*

## Materials and Methods

### Solid-Phase Peptide Synthesis

The peptides were synthesized by the Fmoc-polyamide method on a PS3 peptide synthesizer (Protein Technologies, Inc., AZ, United States) (Lin et al., 2016). Fmoc-amino acid derivatives (0.4 mmol) (AnaSpec, CA, United States) and *O*-(benzotriazol-1-yl)-*N,N,N′,N′*-tetramethyluronium hexafluorophosphate (HBTU) (0.4 mmol) were coupled on Rink Amide AM resin (0.1 mmol) (200–400 mesh, Novabiochem, Germany) in a solution of *N*-methyl morpholine/dimethyl sulfoxide/dimethylformamide (DMF) (4.45/25/70.55% v/v/v). The Fmoc group deprotection step was performed using 30% (v/v) piperidine in DMF. N-terminal acetylation, butyrylation, hexanoylation, octanoylation, myristylation, palmitoylation, and stearylation were performed using four equivalents of acetic anhydride, butyric acid, hexanoic acid, octanoic acid, myristic acid, palmitic acid, or stearic acid, respectively, instead of an Fmoc-amino acid derivative in the final synthetic step. Side chain deprotection and peptide cleavage from the resin were performed by stirring the resin in a cleavage cocktail containing trifluoroacetic acid/water/ethanedithiol (95/2.5/2.5% v/v/v) at room temperature for 2 h. The resin was then removed by passing the reaction mixture through a G2 glass funnel. The peptides in the filtrate were precipitated by adding ten volumes of ice-cold methyl t-butyl ether (MTBE). The precipitate was collected by centrifugation at 3,000 *g* for 15 min at 4°C, washed twice with ice-cold MTBE and dried under vacuum. The crude peptides were purified by reversed-phase high-performance liquid chromatography (RP-HPLC) using a C18 column (10 mm × 250 mm, 10 μm, SUPELCO, Sigma-Aldrich, Germany) and identified by matrix-assisted laser desorption ionization time-of-flight (MALDI-TOF) mass spectrometry (AutoFlex III smartbeam, Bruker, United States). The eluted peptide solution was collected, lyophilized and stored in a −30°C freezer. To prepare the peptide stock solution, the lyophilized peptide powder was dissolved in deionized water, filtered through a 2-μm syringe filter to remove any aggregated peptide, and then quantified by its UV absorption at 280 nm (ε of tryptophan = 5,690 M^–1^ cm^–1^). The peptide stock solution can be stored at −30°C for approximately 4 months.

### Determination of the Minimal Inhibitory/Bactericidal Concentrations

Following CLSI guideline M07-A11, single bacterial colonies of *A. baumannii* ATCC 17978 grown on Müller–Hinton agar (MHA) plates were picked and inoculated in 4 mL of Müller–Hinton broth (MHB) at 37°C and shaken at 180 rpm for 4∼6 h. The bacterial broth was diluted in the same medium to give a cell density of 1∼2 × 10^8^ CFU/mL (OD_600_ = 0.38∼0.4 for *A. baumannii*) (Supplementary Figure 1; Dillon et al., 2020). Then, the broth was diluted 20-fold. Peptides were dissolved in water and filtered through a 0.2-μm filter to make a stock solution. The peptide concentration was quantified by the UV_280_. Then, the peptide solution was serially diluted in MHB to final peptide concentrations of 1, 2, 4, 8, 16, and 32 μg/mL. One hundred microliters of serially diluted peptide and 10 μL of bacterial culture were mixed in a 96-well polystyrene plate. Each well contained approximately 5 × 10^5^ CFU/mL. The positive control was a mixture of 100 μL of MHB and 10 μL of the bacterial culture. The negative control was 110 μL of MHB. The plates were incubated at 37°C without shaking. The minimal inhibitory concentration (MIC) was determined as the lowest concentration of peptide at which no visible bacterial growth occurred after incubation in MHB for 20 h. The bacterium-peptide mixtures (the mixture without visible growth and the mixture containing a two-fold lower peptide concentration than the mixture without growth), positive control, and negative control (3 μL each) were spotted on an MHA plate and incubated at 37°C for 24 h. The MBC was determined as the lowest peptide concentration at which no colonies formed.

### Peptide Stability in Plasma

EDTA-treated rat whole blood was centrifuged at 4°C and 840 × *g* for 5 min, and then the blood cells were removed (Mwangi et al., 2019). The supernatant was placed in an Eppendorf tube and centrifuged at 4°C and 13,000 rpm for 10 min to remove lipids (white precipitate). The supernatant was filtered through a 0.2-μm filter.

The peptide was dissolved in water and filtered through a 0.2-μm filter. The peptide concentration was quantified by the UV_280_ to make a 1 mg/mL stock solution.

Fifteen microliters of peptide solution were mixed with 10 μL of plasma at room temperature for 1 to 72 h. At the indicated time, the peptide was analyzed by HPLC with a C18 column using a linear gradient of 20–65% B over 15 min. Solution A: 5% acetonitrile plus 0.1% trifluoroacetic acid in water; solution B: 0.1% trifluoroacetic acid in acetonitrile.

### Hemolytic Assay

EDTA-treated rat whole blood was centrifuged at 840 × *g* for 3 min at 4°C to separate the blood cells from the plasma, which was removed (Zeitler et al., 2013). The blood cells were washed three times with PBS, where the volume of PBS was the same as that of the original blood, by gently turning the centrifuge tube upside down. After washing, ten microliters of blood cells were diluted 2,000-fold in PBS to count the cells. Finally, red blood cells were diluted to 5 × 10^8^ cells/mL in PBS.

Peptides were dissolved in water and filtered through a 0.2-μm filter to make a stock solution. The peptide concentration was quantitated by the UV_280_. The peptide solution was diluted in PBS to different concentrations (five times the tested concentrations). Twenty microliters of the peptide in PBS was mixed with 80 μL of red blood cell solution in a 96-well plate (V-bottom). The final peptide concentration was 16–256 μg/mL. In the positive control group, 20 μL of 5% Triton X-100 was added to the red blood cell solution for a final Triton X-100 concentration of 1%. For the negative control group, 20 μL of PBS solution was added. The 96-well plate was incubated at 37°C for 45 min. After centrifuging the 96-well plate at 1,500 × *g* for 5 min, 30 μL of the supernatant was mixed with 100 μL of deionized water in a 96-well plate (flat bottom). The concentration of heme in each well was measured by the UV_405_ using an Infinite M1000 pro (Tecan Austria GmbH, Austria). The hemolytic activity was calculated using the following formula.


$$\mathrm{Hemolytic}\,\mathrm{activity}=\left[\left(F-F_{0}\right)/\left(F_{t}-F_{0}\right)\right]\times 100\%$$

F is the UV_405_ of the peptide-treated sample

F_0_ is the UV_405_ of the sample without peptide (negative control)

F_*t*_ is the UV_405_ of the Triton-treated sample (positive control)

### Cell Viability Assay

Cell viability assays were performed by using the CellTiter 96^®^ AQ_*ueous*_ Non-Radioactive Cell Proliferation Assay (Promega, United States), in which 3-(4,5-dimethylthiazol-2-yl)-5-(3-carboxymethoxyphenyl)-2-(4-sulfophenyl)-2H-tetrazolium salt (MTS) can be reduced to purple-colored formazan by intact cells. HEK293 cells in DMEM with 10% FBS were seeded in a 96-well plate (200 μL; cell density 1.25 × 10^5^ cells/mL) and incubated overnight (Lin et al., 2013). The test peptide was dissolved in water and filtered through a 0.2-μm filter. The peptide concentration was quantitated by the UV_280_ to make a 5,120 μg/mL stock solution and then serially diluted in serum-free DMEM. On the second day, the medium was replaced with 100 μL of serum-free DMEM containing different peptide concentrations and incubated for 1 or 24 h. Serum-free DMEM without peptide was used as a negative control and medium containing 1% Triton X-100 was used as a positive control. One milligram of MTS powder was dissolved in 0.5 mL of Dulbecco’s phosphate-buffered saline (DPBS). The MTS solution was mixed with one-twentieth of a phenazine methosulfate (PMS) solution (0.92 g/mL). To each well, 20 μL of the MTS/PMS solution was added. After 3 h, the absorbance was measured with an Infinite M1000 pro (Tecan Austria GmbH, Austria) at a wavelength of 490 nm. Cell viability was calculated using the following equation.


$$\mathrm{Cell}\,\mathrm{viability}=\left[\left(A-A_{p}\right)/\left(A_{n}-A_{p}\right)\right]\times 100\%$$

A is the UV_490_ of the peptide-treated sample

A_*n*_ is the UV_490_ of the sample without peptide (negative control)

A_*p*_ is the UV_490_ of the Triton-treated sample (positive control)

### Time-Kill Kinetics Assay

The time-kill kinetics measurement against *A. baumannii* (ATCC 17978) in blood was conducted following a protocol modified from the literature (Nagarajan et al., 2019). Sixty-six microliters of *A. baumannii* culture (OD_600_ ∼1.2; 3∼6 × 10^8^ CFU/mL in MHB) was diluted in 1,930 μL of rat whole blood (EDTA-treated). Then, 4 μL of the peptide stock solution to be tested (2,035 μg/mL in water) was added to make the final bacterial cell number 10^7^ CFU/mL and the final peptide concentration 4 μg/mL (assigned as tube A). At 2, 4, 6, 8, 10, 20, 30, 40, 50, and 60 min, 100 μL of the bacteria/blood mixture was removed from tube A and mixed with 100 μL of sterilized 2 M NaCl/50% glycerol (twofold dilution, assigned as tube B). Tube B at time points within 10 min was flash-frozen in liquid nitrogen to stop AMP activity since we did not have enough time to perform the following dilution. Ten microliters of the mixture from tube B were mixed with 990 μL of sterilized 1 M NaCl (200-fold dilution, assigned as tube C). Ten microliters of the mixture from tube C was mixed with 90 μL of sterilized 1 M NaCl (2,000-fold dilution, assigned as tube D). Finally, ten or one hundred microliters of solution from each tube was plated on an MHA plate and incubated at 37°C overnight to count the colony number.

To test whether freezing in liquid nitrogen kills part of the bacteria, pepdD2 was tested with and without freezing. To test whether bacteria can be killed by endogenous AMPs in blood, one negative control was set as the same amount of bacterial culture diluted in PBS. Another negative control was set as the same amount of bacterial culture diluted in rat blood without the addition of any peptide. At 0 and 60 min, the bacterial number was measured as described above.

To compare the killing rate of pepI2 and colistin sulfate (Sigma, C4461), incubation was conducted in MHB at 37°C with shaking (180 rpm).

### Biofilm Inhibition Assay

*Acinetobacter baumannii* was cultured in LB overnight at 37°C at 180 rpm and diluted to an OD_600_ of 0.004 (approximately 1∼2 × 10^6^ CFU/mL). Fifty microliters of cell suspension and 50 μL of peptides serially diluted in MHB were added to a growth-enhanced treated 96-well plate (TPP tissue culture plate 96F, Europe, Switzerland) and incubated at 37°C without shaking for 24 h. The positive control was the culture without peptide and the negative control was MHB only. After incubation, the planktonic cells were removed, and then each well was washed with PBS and fixed with 99% methanol for 15 min. Crystal violet solution (0.1% in water) was added to stain the biofilm for 10 min. The wells were washed with water until the negative control (without bacteria) was clear. To resolubilize the crystal violet, 95% ethanol was added, and after 10–15 min, the solubilized crystal violet/ethanol solution was transferred to a new flat-bottomed 96-well plate. The biofilm was quantitated by the absorption of crystal violet at 600 nm using Infinite M1000 pro (Tecan Austria GmbH, Austria) (Mwangi et al., 2019).

### Biofilm Eradication Assay

*Acinetobacter baumannii* was cultured in LB overnight at 37°C at 180 rpm and diluted to an OD_600_ of 0.004 (approximately 1∼2 × 10^6^ CFU/mL). Fifty microliters of cell suspension and 50 μL of MHB were added to a growth-enhanced treated 96-well plate (TPP tissue culture plate 96F, Europe, Switzerland) and incubated at 37°C without shaking for 24 h to form a biofilm. The overnight culture was removed and washed carefully with sterilized water. One hundred microliters of serially diluted pepI2 was added to each well (final peptide concentrations of 2, 4, and 8 μg/mL) and incubated at 37°C without shaking for 24 h. The positive control was the culture without pepI2. Broths with or without pepI2 were removed, and each well was washed carefully with sterilized water. To evaluate whether this AMP can permeate into the biofilm and reduce the number of *A. baumannii*, 100 μL of MHB was added into each well and mixed by pipetting 10 times. Then, MHB together with the suspended biofilm was transferred into a tube containing 2.9 mL of MHB. These tubes were incubated at 37°C with vigorous shaking (180 rpm). At 0, 1, 2, 3, and 4 h post-inoculation, 100 μL of bacterial culture were transferred to a flat-bottomed 96-well plate, and the cell density of the cultures was quantified by the absorption at 600 nm using Infinite M1000 pro (Tecan Austria GmbH, Austria) (Mwangi et al., 2019).

### Circular Dichroism Spectroscopy

Following a protocol modified from the literature (Nielsen and Otzen, 2010), two kinds of liposomes, DOPC and POPE/POPG (1:1, w/w), were prepared. DOPC (10 mg) and POPE/POPG (12 mg) were individually dissolved in 1 mL of chloroform/methanol (2/1, v/v) in a glass tube. The solvents were evaporated under a purge of nitrogen gas to form a thin lipid film on the glass surface. The tube was placed in a vacuum overnight to completely obliterate the organic solvent. We used either deionized water or 20 mM phosphate buffer/100 mM NaCl (pH 7) to rehydrate the lipid film as follows. Six hundred microliters of water or buffer was added to the glass tube, and the solution was mixed using an Intelli-mixer (60 rpm, angle of 45 degrees) for 1 h. Then, the mixture was frozen in liquid nitrogen and thawed at 45°C for 5 min. After five freeze-thaw cycles, the liposomes were prepared by extruding the mixture through a polycarbonate filter (with a 200-nm pore size) using an Avanti Mini-Extruder (Avanti Polar Lipids, United States). PepD3 was dissolved in water to generate a stock solution of 1,280 μg/mL. The pepD3 stock solution was mixed with an equal volume of water or 2 × buffer (40 mM phosphate buffer/200 mM NaCl, pH 7) to make working solutions (peptide concentration of 640 μg/mL). Twenty microliters of pepD3 working solution, 25 μL liposomes, and 155 μL of water or buffer were mixed (final peptide concentration of 64 μg/mL) for CD measurement. CD spectra between 190 and 260 nm were recorded on a J-815 CD spectrometer (JASCO, Japan). The bandwidth was set to 1 nm, and the step resolution was 0.5 nm. Each sample was scanned twice to obtain the final CD spectrum.

### Lipid Composition Analysis

Bacterial lipids were extracted by a modified Folch extraction method (Folch et al., 1957; Jan et al., 2016). Bacterial cells (10^9^ CFU) were collected by centrifugation at 4,000 rpm and 4°C for 10 min. The cell pellet was washed with PBS and then resuspended in 600 μL of methanol/chloroform (2:1, v/v) in an Eppendorf tube. The tube was sonicated in a water bath for 30 min, centrifuged at 13,000 rpm at room temperature for 1 min, and the supernatant (S1) was removed. The debris was resuspended in 600 μL of methanol/chloroform (1:2, v/v) and then sonicated and centrifuged as mentioned above. The supernatant was collected and mixed with supernatant S1. The combined supernatant was mixed with 240 μL of 0.9% KCl. The bacterial lipid was in the lower layer. The lower layer was carefully collected and dried by vacuum centrifugation (Genevac DUO). The dry lipid was dissolved in 20 μL of chloroform/methanol (1:1, v/v) and subjected to thin-layer chromatography (TLC). Briefly, 1 μL of the lipid mixture and/or 35 μL of standard lipids were loaded onto a silica gel TLC (5-cm width × 10-cm length, Silica gel 60 F_254_, Merck) and developed with chloroform/methanol/water (7:3:0.5, v/v/v). Spots were visualized with cerium molybdate stain (10% sulfuric acid, 2.5% ammonium molybdate, and 1% ceric ammonium sulfate aqueous solution) and heated to 90°C. Finally, the lipids on the TLC plate were identified by comparing their Rf values with those of standard lipids. The following lipids were used as standard lipids: 1,2-dipalmitoyl-sn-glycero-3-phosphoglycerol [0.5 mM, PG(16:0)_2_, Sigma], 1,2-dipalmitoyl-sn-glycero-3-phosphoethanolamine [0.5 mM, PE(16:0)_2_, Sigma], 1,2-dipalmitoyl-sn-glycero-3-phosphocholine [0.5 mM, PC(16:0)_2_, Sigma], 1,2-dimyristoyl-sn-glycero-3-phosphoserine [0.5 mM, PS(14:0)_2_, Sigma], and 1,2-dipalmitoyl-sn-glycero-3-phosphate [0.5 mM, PA(16:0)_2_, Sigma]. The lipid extraction was repeated twice, and the TLC separation was conducted three times. The composition of each lipid was normalized to the total lipid intensity on the TLC plate.

## Results

**FIGURE 1:**
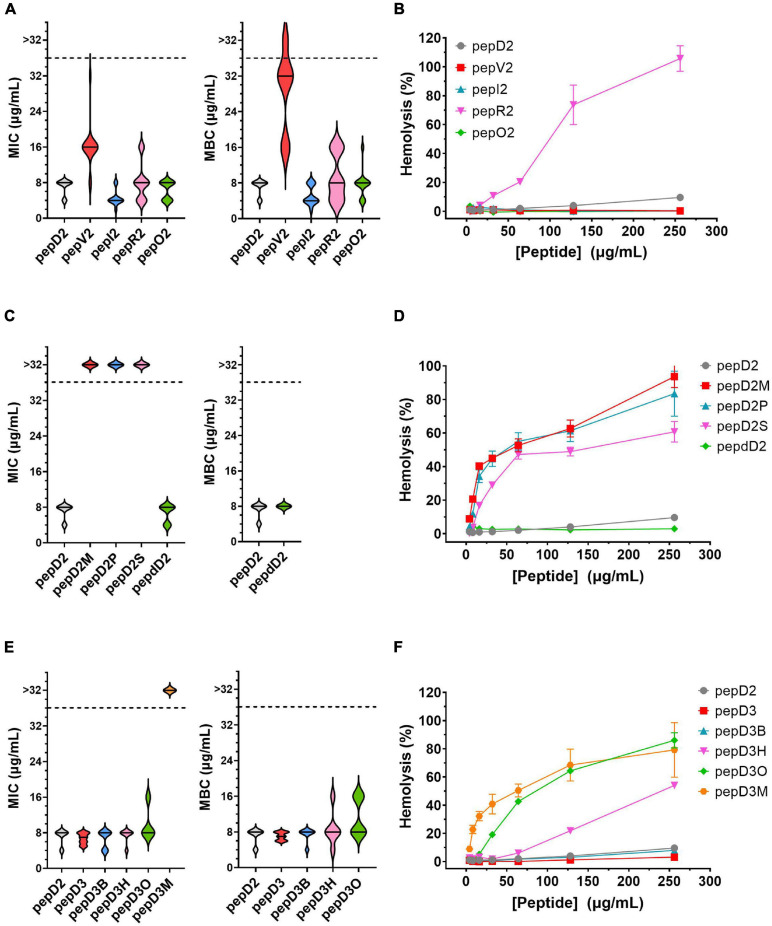
The effect of amino acid substitutions and different N-terminus modifications on the antimicrobial activity and hemolytic activity of the AMPs. Upper panel: changing hydrophobic and positively charged residues; middle panel: using different fatty acyl modifications and D-amino acids; lower panel: using short chain-acyl groups for N-terminal modification. **(A,C,E)** The MIC and MBC values against *A. baumannii* 17978 displayed as a violin plot. Each concentration was tested 3–4 times, and each time, three repeats were used. The median of the data is displayed as a line. **(B,D,F)** Hemolytic activity against rat red blood cells. Each peptide concentration was tested in triplicate. The data are expressed as the mean ± SD.

The MIC and MBC of our designed peptides against *A. baumannii* were obtained by using the broth microdilution method in the CLSI protocol. The bacterial cell number was carefully counted, and a final cell density of 5 × 10^5^ CFU/mL in each well was used in the MIC test (Supplementary Figure 1). The minimal peptide concentration at which no bacterial growth was observed was taken as the MIC value. The culture after peptide treatment was spotted on an MHA plate, and the peptide concentration that could kill all the bacteria was defined as the MBC. The MIC/MBC and hemolytic activity of these peptides are shown and compared in Figure 1.

### Substitution of Hydrophobic and Positively Charged Residues

Both the MIC and MBC of pepD2 against *A. baumannii* were 8 μg/mL. When Leu was replaced by Val, the bactericidal activity of pepV2 was significantly attenuated (Figure 1A). In contrast, when Ile was used instead, the bactericidal activity of pepI2 increased. Changing the positively charged residue from Lys to Arg or Orn did not affect the antimicrobial activity. However, unexpectedly, the hemolytic activity was greatly increased when Arg was used (Figure 1B). In contrast, pepV2, pepI2 and pepO2 had no detectable hemolysis at peptide concentrations up to 256 μg/mL (the corresponding red, green, and blue symbols overlap well in Figure 1B). PepI2 (using Ile instead of Leu) and pepO2 (using Orn instead of Lys) have superior or similar bactericidal activity but lower hemolytic activity than pepD2, making them better candidates for clinical application.

### Influence of D-Form Amino Acids

One major obstacle hampering the clinical application of AMPs is peptide stability. The proteolytic mechanism of enzymes has stereospecificity; therefore, a peptide composed of D-form amino acids is resistant to enzyme digestion. Many studies have reported that replacing L-amino acids with their D-isomers did not affect the antimicrobial activity of the D-form peptides (Mohamed et al., 2017), although the peptide cost increased. Notably, the market price of Fmoc-D-Ile-OH is much higher than that of Fmoc-D-Leu-OH. Considering the cost of D-amino acids, we redesigned pepD2 to a D-form peptide, named pepdD2, in which Lys and Leu were replaced by their D-form isomers while Trp remained as an L-amino acid. Our results showed that pepdD2 has the same MIC and MBC as pepD2 (Figure 1C). Surprisingly, the hemolytic activity of pepdD2 was lower than that of pepD2 (Figure 1D). Thus, pepdD2 is a safer choice than pepD2.

### Longer Is Not Better

One important concern for peptide drugs is the price. Most AMPs are produced by solid-phase peptide synthesis. The peptide length is proportional to the synthesis cost. To save synthesis costs, scientists have tried to determine the shortest active sequence from natural AMPs. For example, the MIC of LL-37 against *A. baumannii* 19606 was 16 μg/mL. Removing the first seven residues from LL-37, as in KS-30, yielded a twofold improvement in the MIC against the same strain (MIC 8 μg/mL) (Feng et al., 2013). However, the antimicrobial efficacy decreased eightfold (MIC 256 μg/mL) in peptide KR-12, which contains only 12 residues from the LL-37 sequence. Here, we produced a shorter peptide, pepD3, which has three residues truncated from the C-terminus of pepD2. PepD2 and pepD3 have a similar percentage of positively charged residues (57% for pepD2 and 55% for pepD3). Comparing pepD2 and pepD3, decreasing the peptide length did not significantly affect the bactericidal activity against *A. baumannii* (Figure 1E). Moreover, the hemolytic activity of pepD3 was lower than that of pepD2. At a peptide concentration of 256 μg/mL, the hemolytic activity of pepD3 was only 3.15%, while that of pepD2 was 9.6% (Figure 1F).

### Effect of End Protection Groups

Many AMPs disrupt the membrane by direct interaction with lipid molecules. Some membrane proteins are anchored to the membrane by end modification with a fatty acyl group, such as myristyl or palmitoyl groups. We chose three saturated fatty acids, myristic acid, palmitic acid, and stearic acid, to interact with the free amino group at the N-terminus of pepD2. The resulting peptides pepD2M, pepD2P, and pepD2S, respectively, could not inhibit the growth of *A. baumannii* at concentrations lower than 32 μg/mL (Figure 1C). Moreover, the hemolytic activities of these three peptides were very high, even at low peptide concentrations (Figure 1D). The data suggested that fatty acyl modification increased the interaction between AMPs and the mammalian plasma membrane and reversed the selectivity between bacterial and mammalian membranes.

Colistin, also called polymyxin E, is synthesized by *Bacillus polymyxa* subspecies *colistinus*. It is a cyclic heptapeptide with a tripeptide side chain acylated by an 8-carbon (colistin A) or 7-carbon (colistin B) acyl chain (Falagas and Kasiakou, 2006). Due to its nephrotoxicity, it is used as a last-resort antibiotic against multidrug resistant Gram-negative bacteria such as *Acinetobacter* species, *Pseudomonas aeruginosa*, *Klebsiella* species, and *Enterobacter* species. Its bactericidal mechanism relies on binding to the negatively charged lipid A portion of lipopolysaccharides of Gram-negative bacteria. People have tried to develop new polymyxins with lower renal toxicity and an improved therapeutic index (Vaara, 2010, 2013, 2019; Velkov et al., 2010; Brown and Dawson, 2017). Des-fatty acyl polymyxin derivatives displayed substantially reduced antibacterial activity and toxicity, suggesting that the acyl chain is crucial for its killing effect (Katsuma et al., 2009). Here, we increased the chain length of the acyl group at the N-terminus of pepD3 from C2 to C4 (pepD3B), C6 (pepD3H), and C8 (pepD3O) individually to optimize the ratio of bactericidal activity to hemolytic activity. Unlike the long-chain fatty acyl group, the short-chain acyl group did not change the MIC or MBC against *A. baumannii* (Figure 1E). However, when the acyl chain length was increased to 6C (pepD3H), a significant increase in hemolysis was observed (Figure 1F). The hemolytic activity was proportional to the chain length: C8 ≫ C6 ≫ C4 > C2 (Figure 1F).

### Peptide Stability

The half-life of a peptide *in vivo* determines its bioavailability. In addition to renal clearance, there are many proteases and peptidases in the body that can degrade AMPs. A short half-life is an important cause of failure of AMPs in clinical applications. Many AMPs that function *in vitro* cannot work properly *in vivo* and can only be applied topically. Common strategies for extending peptide stability include cyclization, modification, and unnatural amino acid and D-amino acid replacement (Mathur et al., 2016). For linear peptides, end protection is the easiest way to protect the peptide from exopeptidase attack. Terminal modification at one end and both ends of a linear peptide Lcf1 (RRWQWR) increased the half-life by twofold and threefold, respectively. Many approved AMPs, such as colistin, daptomycin, and gramicidin, are cyclic peptides that avoid proteolysis. Cyclization extends the half-life of Lcf1 by 48-fold. Many studies have shown that replacing L-amino acids with their D-form isomers did not affect the bactericidal ability of AMPs but extended their half-life in serum (Bessalle et al., 1990; Hamamoto et al., 2002). For example, incorporation of D-amino acids increased the half-life of KSL approximately fourfold (Mathur et al., 2016). Additionally, replacing arginine with α-amino-3-guanidino-propionic acid (Agp) protected the peptide Sub3 from fast degradation in serum (Knappe et al., 2010).

**FIGURE 2:**
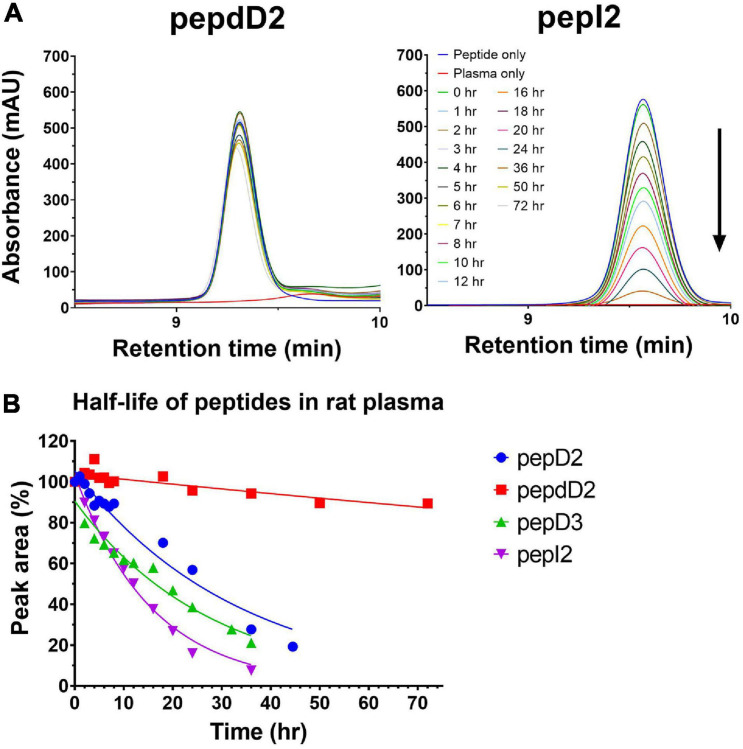
Stabilities of pepD2, pepdD2, pepD3, and pepI2 in rat plasma. **(A)** HPLC profiles of pepdD2 and pepI2 incubated in rat plasma for the indicated times are shown as examples. **(B)** Percentages of intact peptides after different incubation times.

Considering efficacy and safety, the four most effective peptides, pepD2, pepD3, pepI2, and pepdD2, were selected to evaluate how the residue variations affect peptide stability. We used reversed-phase HPLC to examine peptide integrity in plasma over time. The results are shown in Figure 2. Unsurprisingly, pepdD2 had the highest stability. Nearly 90% of the peptide remained intact after incubation in rat plasma for 3 days. PepD3 was degraded faster than the others in the first 6 h. PepI2 was the most effective peptide in killing *A. baumannii*, but its half-life was the shortest (11.18 h) among all the tested peptides.

### Cytotoxicity of AMPs

**FIGURE 3:**
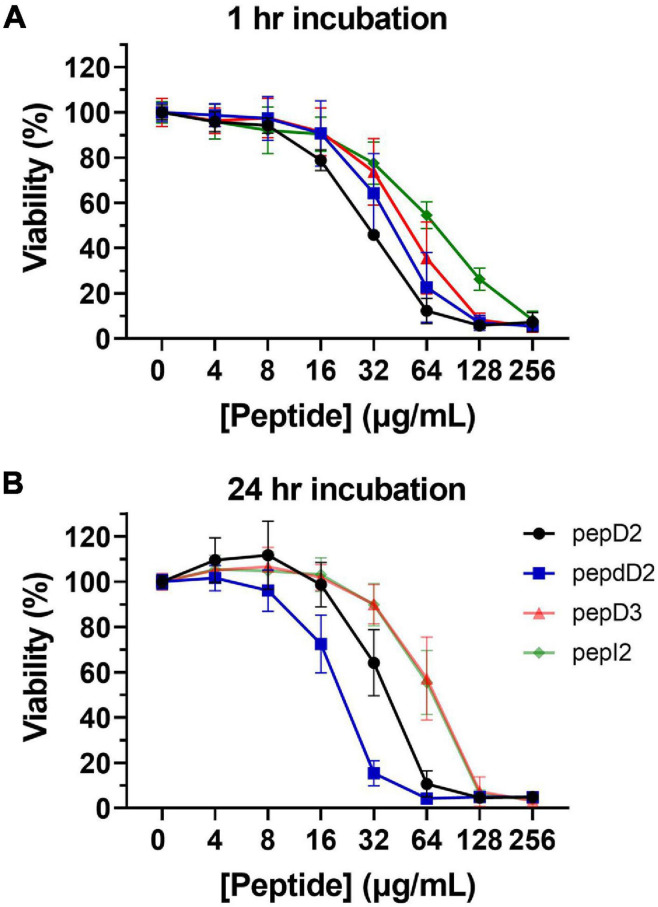
HEK 293 cytotoxicity from pepD2, pepdD2, pepD3, and pepI2. **(A)** 1-h treatment and **(B)** 24-h treatment. The pepD3 and pepI2 data in panel **(B)** are too close to be distinguished. Each concentration was tested independently three times, and each time, three repeats were used. The data are expressed as the mean ± SD.

For clinical application, the selectivity between bacteria and mammalian cells is another important consideration. In addition to the hemolytic assay, the toxicity of the four best peptides, pepD2, pepD3, pepI2, and pepdD2, to HEK293 cells after short-term (1 h) and long-term (24 h) treatment was studied (Figure 3). Notably, pepI2 had the best antimicrobial activity (Figure 1A) and the lowest cytotoxicity (Figure 3). Its IC_50_ in HEK293 cells was approximately 68 μg/mL. Its selectivity index (SI) between HEK293 cells and *A. baumannii* was more than 17. Its SI between RBCs and *A. baumannii* was much higher than 64 since 256 μg/mL pepI2 only causes 0.27% hemolysis (Figure 1B). The D-form peptide pepdD2 had the longest half-life and was the most toxic after 24 h of treatment.

### Time-Kill Kinetics Assay

**FIGURE 4:**
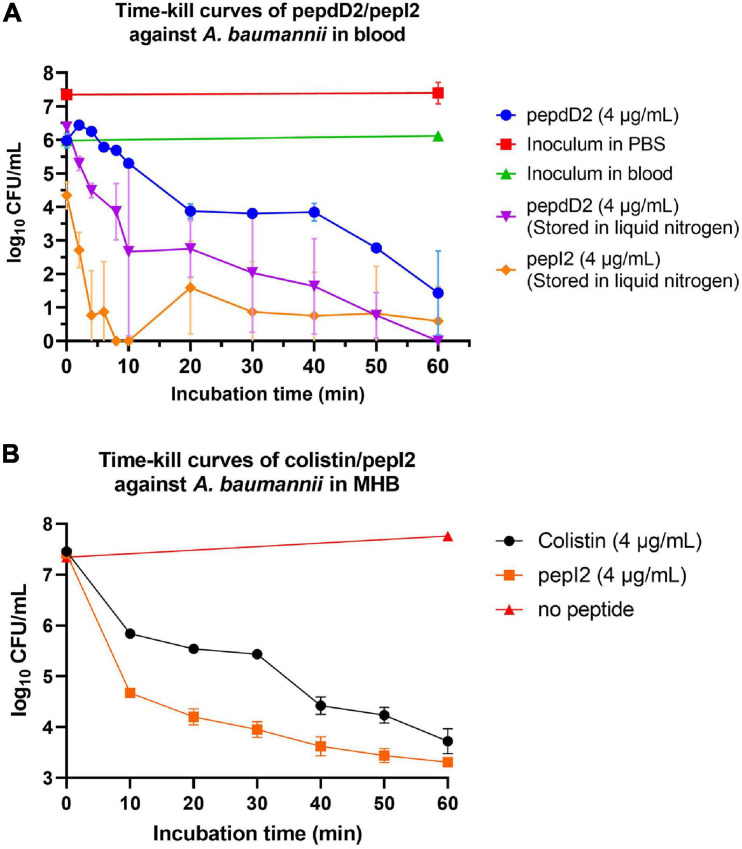
Time-kill kinetics assay. **(A)** Time-kill curves of *A. baumannii* 17978 in blood after treatment with pepdD2 and pepI2. This experiment was performed in *ex vivo* whole rat blood at room temperature without shaking. *A. baumannii* (final concentration of 10^7^ CFU/mL) was incubated with or without peptide (4 μg/mL) for 60 min. At the indicated time points, an aliquot of the sample was removed and diluted, and the remaining cell number was counted by plating on MHA plates. The data are expressed as the mean ± SD. **(B)** The time-kill curves of *A. baumannii* 17978 after treatment with pepI2 and colistin sulfate in MHB. *A. baumannii* (final concentration of 10^7^ CFU/mL) was incubated with or without peptide at 37°C with shaking. The peptide concentration was 4 μg/mL for both peptides.

In addition to peptide stability *in vivo*, AMPs might lose their activity due to binding to serum proteins in the body such as albumin or lipoprotein. To determine whether our AMPs can function well in the blood, a time-kill kinetic study in whole rat blood was conducted. The results showed that 90% of the inoculum could be eliminated in the blood without adding any peptide (green line in Figure 4A). This elimination is probably due to the blood’s self-defense mechanisms, such as macrophage attack or endogenous AMPs secreted by leucocytes. The D-form peptide pepdD2 and the most effective peptide pepI2 were chosen to study their bactericidal kinetics in *ex vivo* rat blood. Both pepI2 and pepdD2 killed 99.99% of bacteria in the blood within 60 min. When the same amount of each peptide (4 μg/mL) was used, pepI2 killed bacteria faster than pepdD2. This is because this peptide concentration is exactly the MBC of pepI2 but the 1/2 the MBC of pepdD2. The experimental procedure followed a procedure in the literature (Nagarajan et al., 2019), in which the blood samples taken within the first 10 min were frozen before plating because these initial samples could not be diluted and plated within such a short period of time. Notably, the CFU at 10 min was lower than that at 20 min (orange line in Figure 4A). To examine whether freezing might kill some of the bacteria, pepdD2 was tested without the freezing step (blue line in Figure 4). The remaining bacterial number in the blood without freezing was indeed at least 10 times higher than that in the samples that had been frozen before plating on MHA plates (comparison of the blue and purple lines in Figure 4A). A 20-mer L-form peptide Ω76 caused a 5 log_10_-fold CFU reduction in *A. baumannii* in human blood within 10 min at a peptide concentration of 32 μg/mL (eightfold its MBC) (Nagarajan et al., 2019). Our pepI2 had the same killing effect at eightfold lower peptide concentrations.

To exclude the influence of the antimicrobial agents in the blood (endogenous AMPs or leucocytes), the time-kill kinetics of pepI2 were repeated in MHB and compared with the kinetics results from colistin sulfate as shown in Figure 4B. Without peptide, *A. baumannii* continued growing in MHB during the 60 min of incubation. PepI2 killed *A. baumannii* faster than colistin sulfate, which has an MIC of 2 μg/mL against *A. baumannii* (Sato et al., 2018).

### Antimicrobial Activity Is Related to the Lipid Composition of Pathogens

**FIGURE 5:**
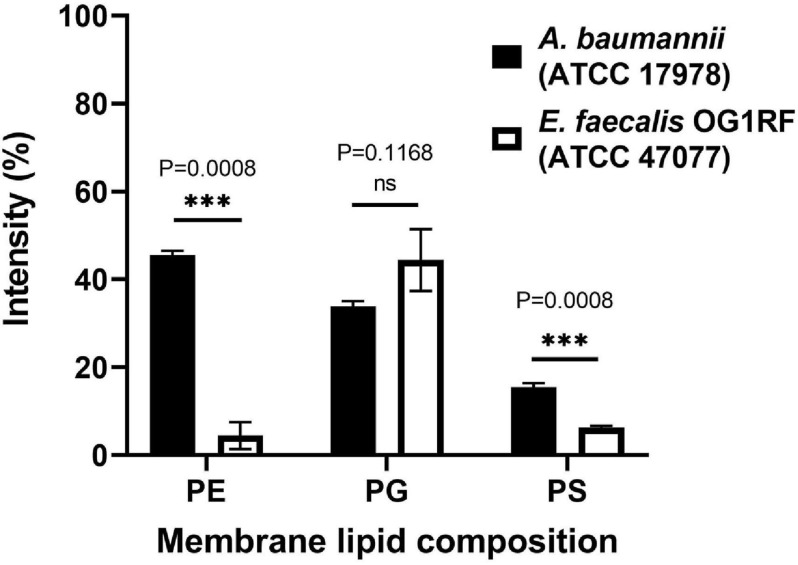
Lipid composition analysis of *A. baumannii* ATCC 17978 and *Enterococcus faecalis* OR1RF ATCC 47077. The lipid composition of the TLC images (Supplementary Figure 4) was analyzed by ImageJ software and normalized to the total lipid intensity. Statistical analysis was performed by Welch’s *t*-test (*N* = 3). ns, not significant, ^∗∗∗^*P* < 0.001.

Unlike polymyxins, our peptide could kill Gram(+) bacteria such as *Staphylococcus aureus* and *Staphylococcus epidermidis* (Supplementary Figure 2), suggesting that our peptides do not function *via* LPS binding. When testing other Gram(+) bacteria, we noticed that our peptides were not effective against *Enterococcus faecalis* at the concentrations used (32 μg/mL and lower). To examine whether the discrimination comes from the bacterial membrane differences, the membrane lipids of these two bacteria were extracted by methanol and chloroform, and TLC was used to analyze the lipid composition (Supplementary Figure 3). The data showed that *E. faecalis* had much lower contents of phosphatidylethanolamine (PE) and phosphatidylserine (PS) than *A. baumannii* (Figure 5).

**FIGURE 6:**
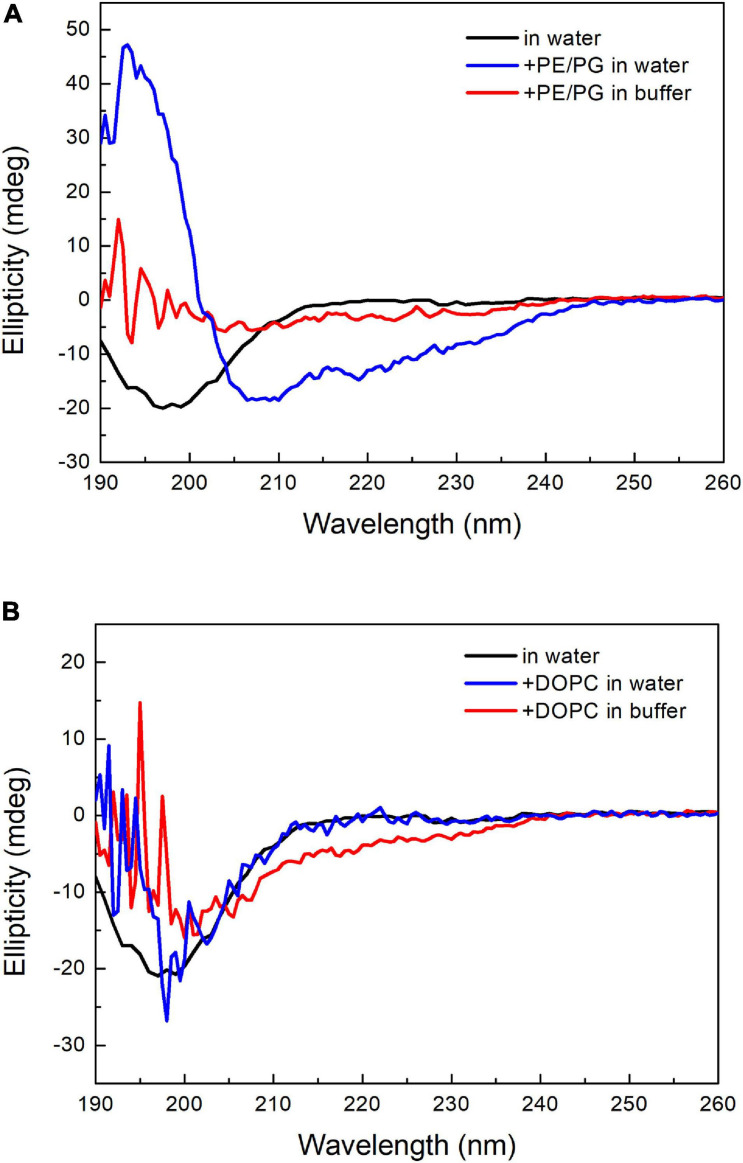
CD spectra of pepD3 interacting with different liposomes. **(A)** PepD3 interacted with PE/PG liposomes prepared in water or in 20 mM phosphate buffer and 100 mM NaCl, pH 7. **(B)** PepD3 interacted with the DOPC liposomes prepared in water or in 20 mM phosphate buffer and 100 mM NaCl, pH 7. The peptide concentration in each sample was 64 μg/mL.

Many AMPs have polycationic charges, which are thought to be important to interact with the negatively charged lipid surface of bacterial membranes, such as phosphatidylglycerol (PG). Leite et al. (2015) reported that anionic PS enhanced the binding of the antimicrobial peptide Polybia-MP1 to the membrane, and PE increased the susceptibility to membrane disruption. Since the major difference in membrane lipid composition between these two bacteria resides in the PE content, we prepared two kinds of liposomes with DOPC and a mixture of POPE/POPG (1:1, w/w). These two liposomes were prepared in either deionized water or 20 mM phosphate buffer/100 mM NaCl (pH 7) to examine the salt effect. The CD spectra of pepD3 interacting with these liposomes are shown in Figure 6. PepD3 was a random coil in water (black line in Figure 6A). When pepD3 was mixed with the PE/PG liposomes in water, it was induced to populate an α-helical structure (blue line in Figure 6A), and the appearance of the sample became turbid. The data suggested that pepD3 disrupted the lipid membranes. When the liposomes were prepared in buffer, the peptide/PE/PG complex aggregated and precipitated at the bottom of the Eppendorf tube, causing the solution to be transparent again. The CD spectrum showed no remaining peptide in the solution (red line in Figure 6A). In contrast, pepD3 could not interact with the DOPC liposomes and remained as a random coil structure (Figure 6B). This experiment indicated that our AMPs disrupt lipid membranes with high PE/PG contents.

Because our AMPs kill bacteria *via* a direct interaction with the membrane, they can efficiently prohibit biofilm formation (Supplementary Figure 4A). However, these peptides could not kill bacteria in the existing biofilms (Supplementary Figure 4B). Our data suggested that the antimicrobial mechanism of our peptides relies on the direct interaction between peptide and lipid just like the lipid precipitation we observed in the peptide/liposome reaction. We surmise that the working mechanism of our AMPs is more likely the carpet model or detergent-like model.

## Discussion

As drug resistance spreads globally and rapidly, AMPs provide a solution to echo the need for novel drugs to defeat pathogens resistant to the currently available antibiotics. AMPs have the advantages of broad-spectrum antimicrobial activity and do not easily induce microbial resistance. However, bioavailability in organisms and the target selectivity between pathogens and host cells are the main reasons that hamper the transition of AMPs from bench to bedside. Currently, only a few AMPs are in clinical trials, and many of them can only be applied topically instead of *via* systemic administration (Fjell et al., 2011; Felício et al., 2017; Magana et al., 2020).

Several groups have designed amphipathic K/L-rich peptides, but many of these peptides have fairly high hemolytic activity and low stability (Blondelle and Houghten, 1992; Papo et al., 2002). A cyclic C(LLKK)_2_C peptide displayed MIC values of 40–75 μg/mL against 20 strains of *A. baumannii.* Intraperitoneal injection twice a day for 3 days rescued half of the mice infected by carbapenem-resistant *A. baumannii* (Huang et al., 2012). In this study, we report a series of *de novo*-designed peptides and the effect of various substitutions and modifications on their antimicrobial efficacy, stability, and safety. Our peptides are linear peptides consisting of 11 or 14 amino acids. They could be easily synthesized by solid-phase peptide synthesis. The properties measured in this study are summarized in Table 2. Replacing four Leu residues with four Val residues increased the MBC value fourfold. Replacing six Lys residues with six Arg residues greatly promotes the hemolysis of red blood cells, suggesting that Arg is the key residue that interacts with the mammalian cell membrane. N-terminus acyl modification could greatly affect hemolytic activity, and the hemolytic activity is proportional to the chain length of the acyl group, where long and hydrophobic fatty acyl chains prefer to interact with the mammalian cell membrane rather than the bacterial cell membrane. In our peptide design, Trp is used to assist peptide quantification. The Trp→Tyr substitution slightly decreased the antimicrobial activity and cytotoxicity (Supplementary Figure 5). In summary, pepI2 is the best AMP with the lowest MIC/MBC values and the highest SI between kidney and bacterial cells. PepdD2 is the most stable AMP in plasma, but its higher cytotoxicity than its parent peptide pepD2 might be due to its stability. Both peptides could eliminate 99.99% of *A. baumannii* in the blood within 1 h at a peptide concentration of only 4 μg/mL. Their mechanism of action is different from that of colistin; hence, they might be useful to combat colistin-resistant strains.

**TABLE 2:** Comparison of the designed AMPs in this study.

Peptide	MIC/MBC *A. baumannii* 17978 (μg/mL)	Hemolysis (%) at 256 μg/mL	HEK293 IC_50_ (μg/mL)		Half-life in plasma (h)	Selectivity index (SI)^a^
		
			1 h	24 h		
pepD2	8/8	9.6	29.1	37.3	23.08	4.7
pepD3	8/8	3.15	49.5	68.1	19.13	8.5
pepV2	16/32	0.31				
pepI2	4/4	0.27	68.5	66.8	10.86	16.7
pepR2	8/8	105.65				
pepO2	8/8	0.23				
pepdD2	8/8	2.9	40.2	20.6	289.6	2.6
pepD2M	>32/x	93.63				
pepD2P	>32/x	83.45				
pepD2S	>32/x	60.70				
pepD3O	8/8	86.03				
pepD3H	8/8	54.07				
pepD3B	8/8	7.89				

*“x” indicates that the MBC could not be measured due to an MIC > 32 μg/mL. Only four peptides with a low MBC and low hemolysis had their cytotoxicity and half-life measured. ^*a*^SI is equal to the HEK293 IC_50_ (24-h treatment) divided by MBC.*

## Data Availability Statement

The raw data supporting the conclusions of this article will be made available by the authors, without undue reservation.

## Ethics Statement

The animal study was reviewed and approved by Institutional Animal Care Use Committee, Academia Sinica.

## Author Contributions

S-PC synthesized peptides and conducted most of the experimental work and data analysis. EC studied the peptide/liposome interaction. S-YY performed the biofilm and time-kill assay. P-SK assisted the time-kill assay. H-MJ, T-CY, M-YH, K-TL, and C-HL assisted lipid composition analysis. RC designed the experiments, analyzed data, and wrote the manuscript. All authors contributed to the article and approved the submitted version.

## Conflict of Interest

The authors declare that the research was conducted in the absence of any commercial or financial relationships that could be construed as a potential conflict of interest. A provisional patent application (63/117,530) has been filed.

## Supplementary Material

Supplementary Figure 1 Plots of cell number vs. optical density at 600 nm of bacteria in MHB medium.

Supplementary Figure 2 MIC and MBC values of the AMPs against Gram-positive*Staphylococcus aureus* and *Staphylococcus epidermidis.*

Supplementary Figure 3 Representative TLC image of the extracted bacterial membrane lipids of *A. baumannii* and *Enterococcus faecalis.*

Supplementary Figure 4 Biofilm inhibition assay and biofilm eradication assay.

Supplementary Figure 5 The effect of aromatic residues on the antimicrobialactivity and cytotoxicity of the AMPs.
