# Corrigendum: A Systematic Study of the Stability, Safety, and Efficacy of the *de novo* Designed Antimicrobial Peptide PepD2 and Its Modified Derivatives Against *Acinetobacter baumannii*

**DOI:** 10.3389/fmicb.2021.821347

**Published:** 2022-01-14

**Authors:** Sung-Pang Chen, Eric H-L Chen, Sheng-Yung Yang, Pin-Shin Kuo, Hau-Ming Jan, Tsai-Chen Yang, Ming-Yen Hsieh, Kung-Ta Lee, Chun-Hung Lin, Rita P-Y Chen

**Affiliations:** ^1^Institute of Biological Chemistry, Academia Sinica, Taipei, Taiwan; ^2^Institute of Biochemical Sciences, National Taiwan University, Taipei, Taiwan; ^3^Department of Biochemical Science and Technology, National Taiwan University, Taipei, Taiwan

**Keywords:** antimicrobial peptide, antibiotic-resistant, *Acinetobacter baumannii*, lipid, membrane, drug-resistant

In the original article, there was an error. “Gram(–) bacteria” was written where “Gram(+) bacteria” should have been used.

A correction has been made to **Results, Antimicrobial Activity is Related to the Lipid Composition of Pathogens**, paragraph 1:

“Unlike polymyxins, our peptide could kill Gram(+) bacteria such as *Staphylococcus aureus* and *Staphylococcus epidermidis* (Supplementary Figure 2), suggesting that our peptides do not function *via* LPS binding. When testing other Gram(+) bacteria, we noticed that our peptides were not effective against *Enterococcus faecalis* at the concentrations used (32 μg/mL and lower). To examine whether the discrimination comes from the bacterial membrane differences, the membrane lipids of these two bacteria were extracted by methanol and chloroform, and TLC was used to analyze the lipid composition (Supplementary Figure 3). The data showed that *E. faecalis* had much lower contents of phosphatidylethanolamine (PE) and phosphatidylserine (PS) than *A. baumannii* (Figure 5).”

The authors apologize for this error and state that this does not change the scientific conclusions of the article in any way. The original article has been updated.

## Publisher's Note

All claims expressed in this article are solely those of the authors and do not necessarily represent those of their affiliated organizations, or those of the publisher, the editors and the reviewers. Any product that may be evaluated in this article, or claim that may be made by its manufacturer, is not guaranteed or endorsed by the publisher.

