# Supplementary material for: A Systematic Study of the Stability, Safety, and Efficacy of the de novo Designed Antimicrobial Peptide PepD2 and Its Modified Derivatives Against Acinetobacter baumannii
Source: Front Microbiol. 2021 Jun 18;12:678330. doi: 10.3389/fmicb.2021.678330 (PMC8250858; doi:10.3389/fmicb.2021.678330)
Supplement: Supplementary Figure 1 — Plots of cell number vs. optical density at 600 nm of bacteria in MHB medium. [file Data_Sheet_1.pdf]

A systematic study of the stability, safety, and efficacy of the de novo designed antimicrobial peptide pepD2 and its modified derivatives against *Acinetobacter baumannii*

Sung-Pang Chen[1,2], Eric, H.-L. Chen[1], Sheng-Yung Yang[1], Pin-Shin Kuo[1,2], Hau-Ming Jan[1], Tsai-Chen Yang[1], Ming-Yen Hsieh[1], Kung-Ta Lee[3], Chun-Hung Lin[1,2], Rita P.-Y. Chen[1,2]

[1]Institute of Biological Chemistry, Academia Sinica, Taipei 11529, Taiwan

[2]Institute of Biochemical Sciences, National Taiwan University, Taipei 10617, Taiwan

[3]Department of Biochemical Science and Technology, National Taiwan University, Taipei 10617, Taiwan

Supplementary Materials

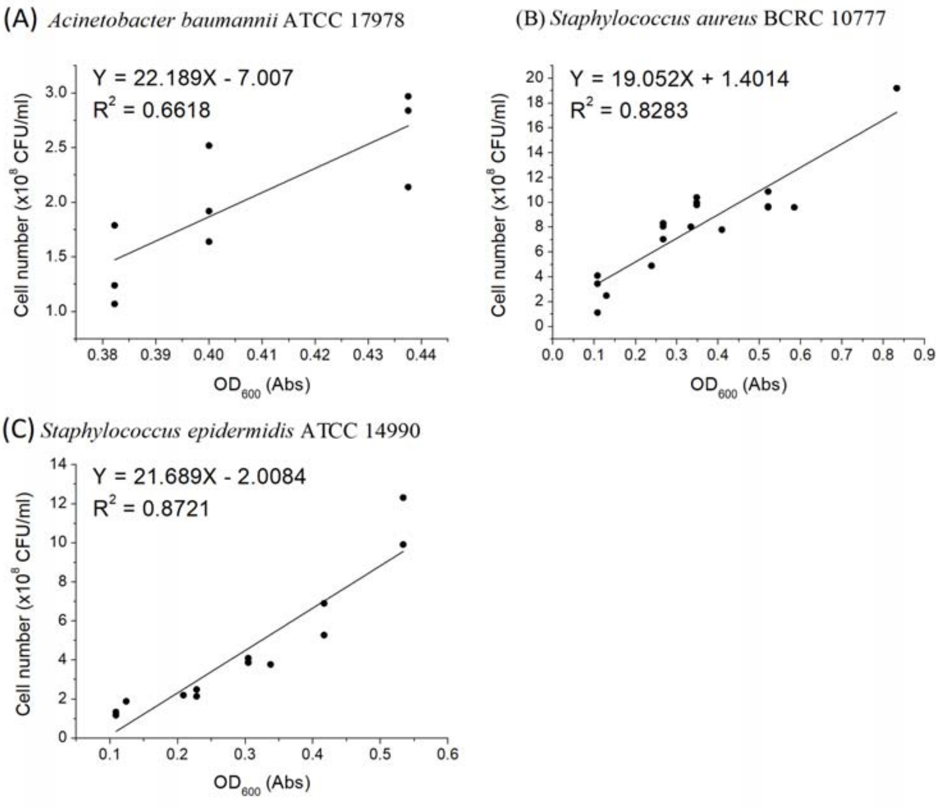

Fig. S1. Plots of cell number versus optical density at 600 nm of bacteria in MHB medium. (A) *Acinetobacter baumannii* ATCC 17978, (B) *Staphylococcus aureus* BCRC 10777, and (C) *Staphylococcus epidermidis* ATCC 14990. For the MIC measurement, the bacterial broth was diluted in MHB to give a cell density of 1~2 $\times 10^8$ CFU/mL (OD_{600}=0.38~0.4 for *Acinetobacter baumannii* ATCC 17978; OD_{600}=0.033 for *Staphylococcus aureus* BCRC 10777; and OD_{600}=0.14~0.18 for *Staphylococcus epidermidis* ATCC 14990).

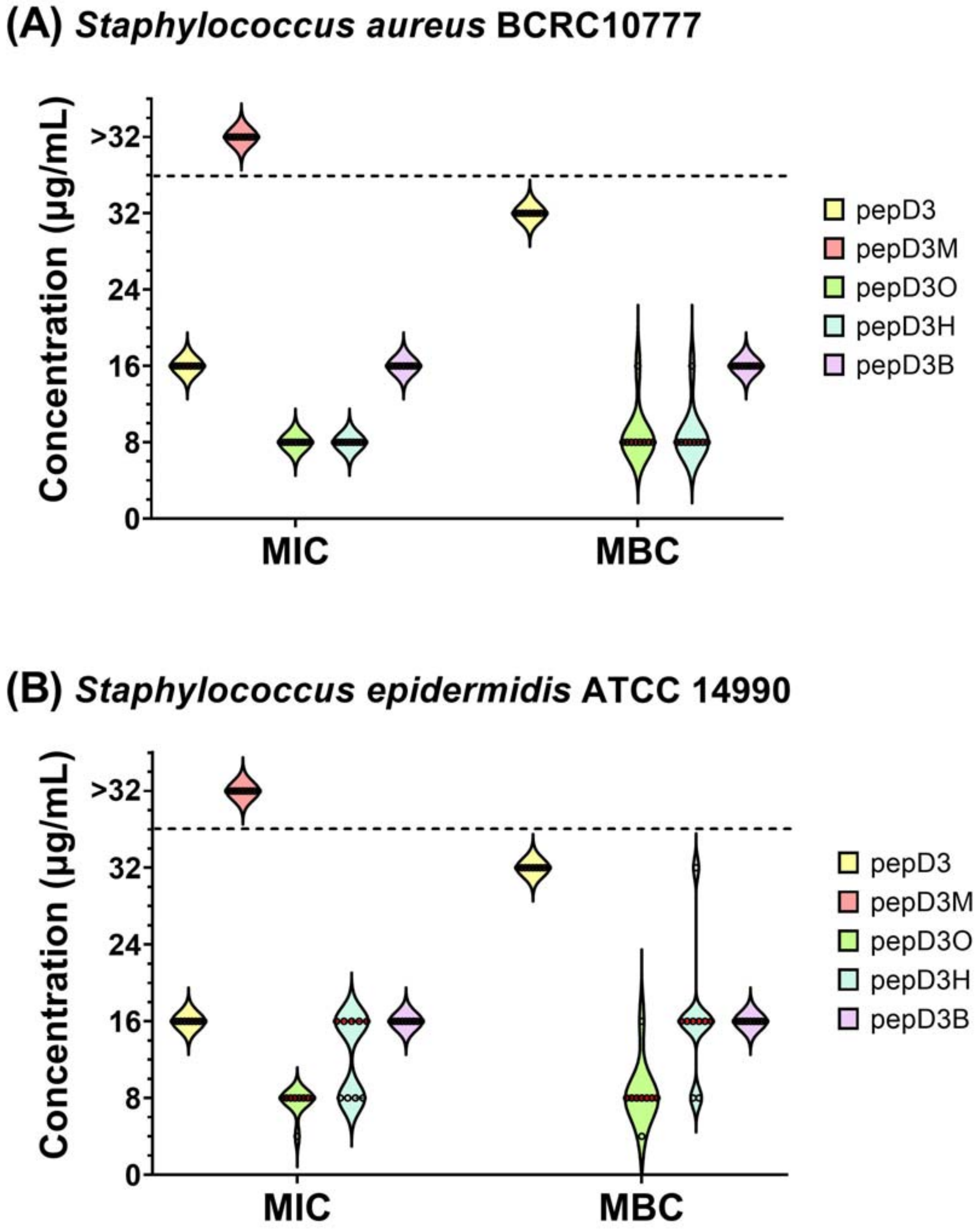

Fig. S2. MIC and MBC values of the AMPs against gram-positive *Staphylococcus aureus* and *Staphylococcus epidermidis*.

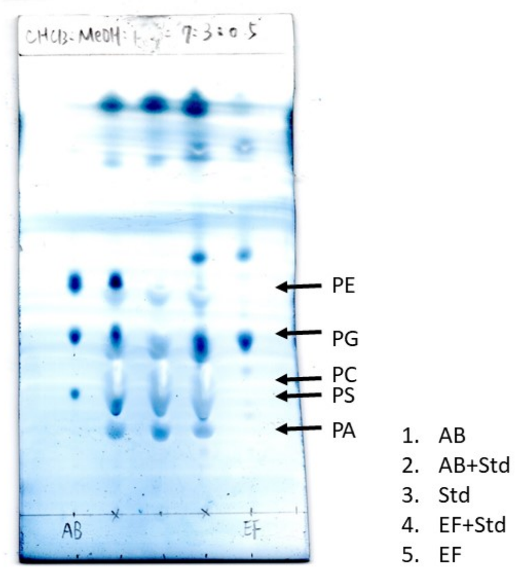

Fig. S3. Representative TLC image of the extracted bacterial membrane lipids of *A. baumannii* ATCC 17978 and *Enterococcus faecalis* OR1RF ATCC 47077. 1, *A. baumannii*; 2, *A. baumannii* + standard lipids; 3, standard lipids; 4, *E. faecalis* + standard lipids; and 5, *E. faecalis*.

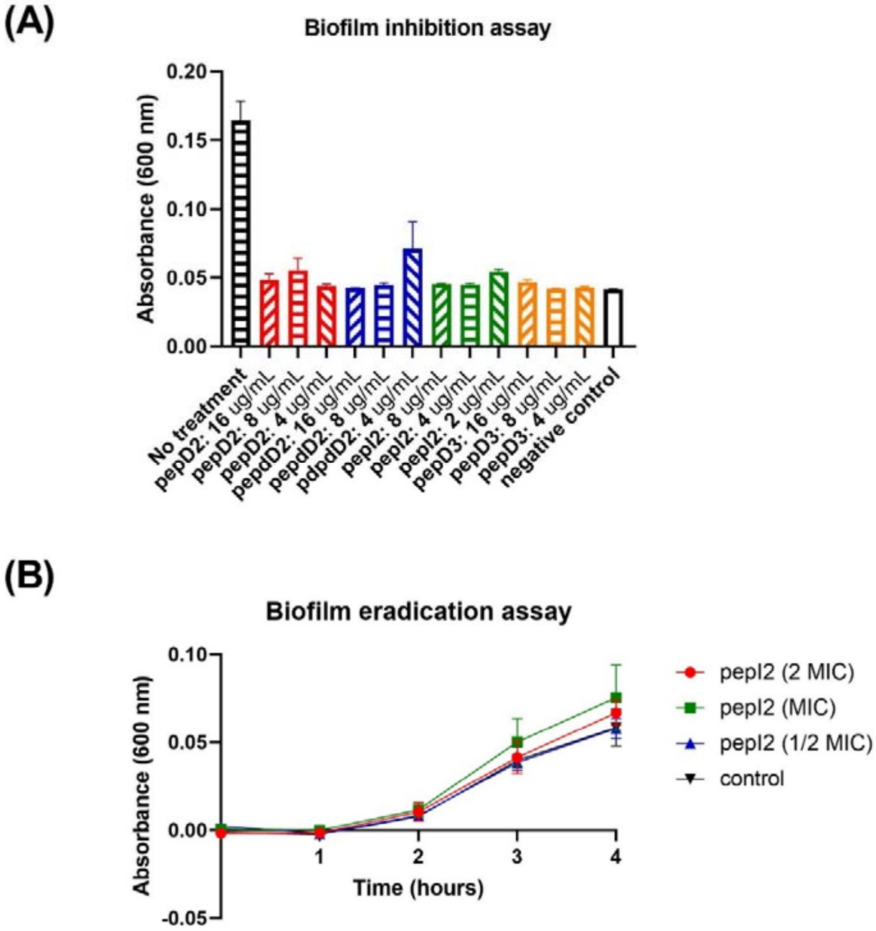

Fig. S4. (A) Biofilm inhibition assay of pepD2, pepdD2, pepI2, and pepD3 against *Acinetobacter baumannii* ATCC 17978. Three peptide concentrations (1/2 MIC, MIC, and 2× MIC) of each peptide were tested. The bacterial culture without the addition of any peptide is marked as "No treatment". The negative control was LB broth without bacteria. (B) Biofilm eradication assays of pepI2. Three peptide concentrations, 1, 2, and 4 μg/mL (1/2 MIC, MIC, and 2× MIC), of pepI2 were tested. The biofilm without peptide treatment is marked "control". N=3 for both assays.

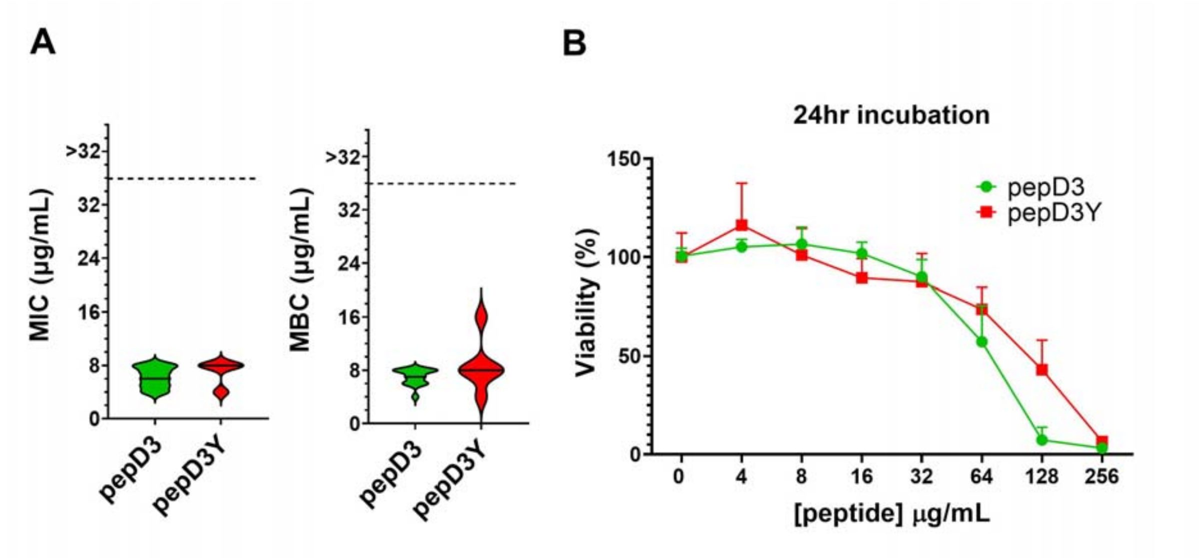

Fig. S5. The effect of aromatic residues on the antimicrobial activity and cytotoxicity of the AMPs. PepD3 sequence: Ac-WKKLKKLLKKL-NH_2; pepD3Y sequence: Ac-YKKLKKLLKKL-NH_2. (A) MIC and MBC against *A. baumannii* 17978. Each concentration was tested 3-5 times, and each time, at least 3 repeats were used. The median of the data is displayed as a line. (B) HEK 293 cytotoxicity. Each concentration was tested independently 3 times, and each time, 3 repeats were used. The data are expressed as the mean ± SD.
